# The Role of Implementation Climate in Moderating Educator Use of Evidence-Based Practices and Outcomes for Autistic Students

**DOI:** 10.1007/s10803-024-06443-x

**Published:** 2024-07-01

**Authors:** Aubyn C. Stahmer, Yue Yu, Jessica Suhrheinrich, Melina Melgarejo, Patricia Schetter

**Affiliations:** 1 Department of Psychiatry and Behavioral Sciences, University of California Davis MIND Institute, Sacramento, CA USA; 2 Department of Special Education, San Diego State University, San Diego, CA USA; 3 Child and Adolescent Services Research Center, San Diego, CA USA; 4 California Autism Professional Training and Information Network (CAPTAIN), Sacramento, CA USA

**Keywords:** Implementation climate, School-based services, Autism, Special education

## Abstract

Ensuring effective use of evidence-based practice (EBP) for autism in schools is imperative due to the significantly increasing number of autistic students receiving school services each year. High-quality EBP use has proven challenging in schools. Research indicates implementation climate, or how EBP are supported, rewarded, and valued, and EBP resources are related to successful implementation. However, limited understanding of system-level contextual factors that impact EBP implementation for school-based providers makes development of appropriate implementation supports challenging. Understanding these factors is crucial for selecting and tailoring implementation strategies to support EBP scale up. In this observational study, California school-based providers (n = 1084) completed surveys related to implementation climate, leadership, autism experience and EBP implementation (use, competence, knowledge). Student outcomes included state level academic and behavioral indicators. Using an implementation science framework (Aarons et al., in Administration and Policy in Mental Health and Mental Health Services Research 38:4–23, 2011) and multilevel modeling, we examined the relationship between EBP Implementation and student outcomes and the moderation effects of provider and district level factors. Higher implementation climate predicted better EBP implementation outcomes, and proved more impactful when provider hands-on autism experience was low. Greater EBP resources predicted a higher percentage of students who met math standards only when district poverty level was high. Our findings suggested moderating effects on EBP implementation from both provider and system level factors. Implementation climate and resources may be especially key in addressing equity issues related to high poverty schools in which teachers often have less autism experience.

## Introduction

The role of implementation climate in moderating educator use of evidence-based practices and outcomes for autistic students.

Implementation of research-informed and evidence-based autism practices (EBP) remains challenging in the complex context of public school systems (Cook & Odom, 2013; Dingfelder & Mandell, 2011). To improve EBP use we need a better understanding of the multiple factors related to the system, school, educator, and student context that impact EBP implementation success. High quality EBP use in schools remains a key factor in providing equal access to appropriate intervention for many students with complex needs, including those with autism. However, while EBP that address a range of learning and behavioral differences have been identified for autistic students for over two decades (Odom et al., 2010; Steinbrenner et al., 2020; Wong et al., 2014, 2015), school implementation of these autism-specific practices remains limited (Kraemer et al., 2019; Odom et al., 2022). Because the number of autistic students is increasing in schools (OSEP, 2024), education systems need to understand the key mechanisms which may affect EBP implementation to increase access and equity to high quality services for all students.

An additional concern relates to equity in access to quality services for autistic students. A recent evaluation of Individual Education Program (IEP) records from 18,000 autistic students found disparities in how autism services were distributed. Black, Hispanic, Asian American, and low-income autistic students received fewer autism-specific school-based services than white and higher-income students (Sturm et al., 2021). This expands on findings from a recent systematic review of 11 articles indicating racial and ethnic minority groups and children from low-income families had less access to acute care, specialized services, educational services, and community services compared with higher-income and white families (Smith et al., 2020). Equitable access to evidence-based services for low-income and racially minoritized children is a critical issue for the field. The *2020 National Indicators Report: Children on the Autism Spectrum and Family Financial Hardship* found that over half of children with autism live in low-income households (household income below 200% of the federal poverty level) and 30% live in very low-income households (Anderson et al., 2020). Understanding how to improve the educational context for improving access to care for all students has the potential to improve outcomes and reduce disparities.

High poverty districts may also lack resources for autism EBP implementation. A qualitative study examining implementation processes for autism EBP use indicated that resources such as access to curricula, manipulatives, classroom space, and professional development could be either a barrier or facilitator to effective implementation (Suhrheinrich et al., 2021). Similarly, a top barrier reported by teachers training to implement social skills intervention with elementary age autistic students included a lack of materials such as training tools, access to on-line resources, reinforcement incentives, and limited resources for training (Silveira-Zaldivar & Curtis, 2019). Understanding how access to resources might interact with other factors, such as teacher experience and implementation climate, may provide clues to providing improved supports to schools with limited resources, thereby improving student outcomes.

Implementation science, or the study of methods support the uptake of EBP into public service systems, provides frameworks to support the understanding of contextual factors that may influence EBP use in community settings (Ogden & Fixen, 2014). Implementation frameworks provide guidance on how to measure and understand organizational, leadership and competency drivers that may facilitate or hinder successful EBP implementation. Understanding these drivers allows researchers to identify strategies to address implementation challenges for specific contexts. Please see Boyd et al., (2022) for a description of how implementation science can be applied specifically to moving autism EBP into practice.

Implementation climate is an organizational driver which may be a key mechanism affecting EBP implementation (Lyon et al., 2018; Turner et al., 2018). Implementation climate refers to the extent to which an innovation or EBP is expected, supported, and rewarded in an organization or system (Weiner et al., 2011). Implementation climate has been linked to high quality, effective use of autism EBP in public schools (Dingfelder & Mandell, 2011; Webster & Roberts, 2020; Williams et al., 2019). High quality, or high fidelity EBP implementation, in turn, predicts child outcomes (Zitter et al., 2021). Autism EBP often address student attention to learning activities and classroom engagement, which in turn, can improve academic outcomes (e.g., Stahmer et al., 2023a, 2023b). In fact, school implementation climate interacts with EBP fidelity such that both strong fidelity and a strong climate for implementation are necessary to ensure students do well in school (Kratz et al., 2019). Additionally, strong implementation climate leads to increased EBP sustainment, decreased staff burnout, and improved child outcomes in public service systems (Ehrhart et al., 2014; Locke et al., 2019; Lyon et al., 2018; Novins et al., 2013). Importantly, implementation climate is a malleable factor that could be a target of an implementation intervention which could facilitate more effective EBP use in schools.

Recently we conducted a statewide survey of over 2000 education system administrators and educators serving autistic students in California. Data indicate that overall implementation climate could be improved, especially at the district and school levels (Stahmer et al., 2023a, 2023b). County and regional education agencies focused on special education had higher implementation climate, but this did not translate to the school level which is where teachers are attempting to implement EBP with autistic students. Participating educators included a range of direct service providers working with autistic students including teachers, specialists who often work with this population (e.g., speech and language therapists; McDonald et al., 2019), and paraprofessionals. We included paraprofessionals because they make spend a large portion of their days implementing behavioral plans and providing instructions to students with disabilities (Giangreco & Broer, 2005; Giangreco et al., 2010) including many students on the autism spectrum (Biggs et al., 2019). Therefore, although paraprofessionals are not independent practitioners, their perspective in implementation climate, implementation resources, and implementation outcome is informative.

In the current study, using an implementation science framework (Aarons et al., 2011), we explored provider and system level moderating factors on provider EBP implementation and student outcomes. We aimed to: (1) examine the relationship between implementation climate and educator reported outcomes of EBP implementation; and (2) examine whether educator experience moderates the influence of implementation climate on EBP implementation. We also included an exploratory third aim to (3) examine the relationship between EBP resources and student outcomes in districts with varying levels of poverty.

## Methods

We have employed the exploration, preparation, implementation, sustainment (EPIS) implementation framework designed for public service sectors (Aarons et al., 2011) to guide our statewide work examining facilitators and barriers to the use of autism EBP. EPIS integrates a multi-level framework to highlight factors influencing implementation including outer (e.g., State and SELPA level climate and structure) and inner (e.g., district and teacher characteristics) contexts. This study examines implementation climate at multiple levels and inner context factors related to district resources and teacher experience.

## Participants

Survey data were collected from California school personnel for the 2018/2019 school year. Participants were school-based providers (n = 1084), representing 333 districts and delivering direct services (e.g., general education teachers, special education teachers, paraprofessionals, speech-language pathologists; See Table 1). The demographics of our sample is similar to public school teachers in California (2018–2019 school year; California Department of Education, 2022b) and in the US (2017–2018 school year; Institute of Education Sciences). The majority of participants (81%) identify as female (compared to 73% in CA, 76% nationwide), 63% had a master’s degree or higher (not available in CA, 58% nationwide), 16% identified as Hispanic (21%, 9%), 71% were White (61%, 79%), 1.4% Black (4%, 7%), 4% Asian (6%, 2%), 3% were two or more races (1%, 2%), 0.6% Native American/Alaska Native (5%, 1%), and 0.6% Pacific Islander (3%, less than 1%). See Table 1.

**Table 1:** Participants characteristics (N = 1083)

	%
Sex	
Female (n = 884)	81.5
Male (n = 120)	11.1
Other (n = 4)	0.40
Education level	
High-school (n = 60)	2.0
AA (n = 158)	5.2
BA (n = 676)	22.3
Master’s (n = 1928)	63.5
Doctorate (n = 142)	4.7
Race	
Native american (n = 7)	.60
Asian (n = 47)	4.3
African american/black (n = 15)	1.4
Native hawaiian or other pacific Islander (n = 7)	.60
White (n = 770)	71.0
More than one race (n = 32)	3.00
Ethnicity	
Hispanic (n = 177)	16.3
Non-hispanic (n = 797)	73.5
Age	
18–24 (n = 20)	1.8
25–44 (n = 552)	50.9
45–64 (n = 395)	36.4
65–74 (n = 25)	2.3
Job titles	
Special education teacher (n = 639)	59.0
Paraprofessional (n = 158)	14.6
SLP/SLPA (n = 93)	8.6
Psychologist/mental health counselor (n = 87)	8.1
Itinerant special education teacher (n = 21)	1.9
General education teacher (n = 17)	1.6
OT/OTA (n = 12)	1.1
Other (e.g., learning specialists, behavioral specialists, school counselors, physical therapists) (n = 11)	1.1
Missing (n = 42)	3.9

*AA* Associate in arts, *BA* bachelor of arts, *SELPA* special education local plan areas, *SLP*/*SLPA* speech language pathologist/speech language pathologist assistant, *OT*/*OTA* occupational therapist/occupational therapist assistant

## Measures

Participants completed surveys about implementation climate (ICS; Ehrhart et al., 2014), provider experience with autism, EBP resources, and EBP implementation outcomes (fidelity, competence, knowledge). Student outcomes related to accountability, academic and behavioral indicators were obtained from the California Department of Education.

## Implementation Climate Scale (ICS)

This study used a combined implementation climate scale (ICS; Ehrhart et al., 2014) and school-implementation climate scale (Lyon et al., 2018; Thayer et al., 2022). The 25-item ICS measures perceptions of the policies, practices, procedures, and behaviors that are expected, rewarded, and supported to facilitate effective EBP implementation in the education system. Participants rated the extent to which they agreed with statements about EBP values and priorities, and items were rated on a 5-point Likert scale (0 = “not at all” to 4 = “very great extent”)*.* The sum of the mean scale scores was used in analyses, ranging from 0 to 100. Normative scores are not available for this measure. The internal consistency reliability is strong for the ICS (*α* = 0.91; Ehrhart et al., 2014) and the S-ICS (*α* = 0.93; Lyon et al., 2018). Direct service providers (DSP; teachers, paraprofessionals, and related service professionals) completed the ICS both on their district and their school site.

## Autism-Related Experience

Participant’s hands-on experience working with students with autism was measured by answering, “rate your level of “hands-on” experience working with students with autism”, which was rated on a 4-point Likert scale (0 = “little to no hands-on/direct experience working with students with autism” to 3 = “extensive hands-on experience working with a student with autism”).

## EBP Resources

The adapted Autism EBP Resources Assessment Tool (Luke et al., 2014) was used to assess resources for autism EBP use. Three subscales were used in this study (partnerships, organizational capacity, and strategic planning). The 15-item survey asked about the cultivation of connections between autism EBP use and stakeholders (*α* = 0.90; Luke et al., 2014), organizational capacity to implement the practices (*α* = 0.87; Luke et al., 2014), and the use of strategic planning to guide goals and strategies related to autism EBP use. (*α* = 0.88; Luke et al., 2014). The items were rated on a 7-point Likert scale (1 = “too little or no extent” to 7 = “to a very great extent”). A total score was used.

## EBP Implementation

Participants answered an adapted version of the evidence-based practice outcomes scale (Ehrhart et al., 2015), which was used to measure the extent to which they (1) use all components of their primary EBP, (2) have adapted their primary EBP, (3) feel competent implementing their primary EBP, and (4) feel knowledgeable explaining their primary EBP. The four-item measure has strong internal consistency reliability (*α* = 0.97; Ehrhart et al., 2015). Participants self-rated on a 5-point Likert scale (0 = “Not at all” to 4 = “Very great extent”). Normative scores are not available for this measure. An average score of the four items was used to examine the relationship to other measures.

## Poverty Rate

The poverty rate of each district was retrieved through publicly available data on California school dashboard (caschooldashboard.org) and was labeled as “socioeconomically disadvantaged”, which is defined as students who are eligible for free or reduced meals or have caregivers who did not receive a high school diploma.

## Student Outcomes

Student outcomes were derived from the California Department of Education for the 2018/2019 school year and are California accountability academic and behavioral indicators that assess how local educational agencies and schools are meeting the needs of their students, including the percentage of students with autism that scored at least Level 3 (Standard Met) in math and english, percentage of students with autism in regular class for greater than 80% of the day, percentage of students with autism in separate placements, and percentage of suspension within the given year. Student data was for Districts in which we had participating educators.

## Statistical Analyses

Descriptive statistics were used to describe the demographic information of the sample. Multilevel modeling was conducted in R using lme4 package (Bates et al., 2015) in order to account for the nested nature of the data, where participants were grouped within Districts which, in turn, were grouped within geographic regions (referred as Special Education Local Plan Area, “SELPA”, in California). We first examined an unconditional model, with no fixed effects, in order to assess the variances of the random effects of District and SELPA. For simplicity, District and SELPA were modeled as orthogonal random effects. As a result, 3.2% of the variance in EBP implementation outcome was accounted for by District, and 1.6% by SELPA. District and SELPA both accounted for very little variance, but were retained in the model as random effects as per the design of the study:

$${\text{Model 1: EBP outcome}}\, \sim \,{1}\,{ + }\,\left( {\text{1|District}} \right)\, + \,{\text{(1|SELPA)}}{.}$$

Building on this base model, we next added implementation climate as a fixed main effect:

$${\text{Model 2: EBP outcome}}\, \sim \,{1}\, + \,{\text{ICSTotal}}\,{ + }\,\left( {\text{1|District}} \right)\,{ + }\,\left( {\text{1|SELPA}} \right){.}$$

To test the 2 models above, the deviance values between-2LogLikelihood scores distributed as a Chi-square, with the degrees of freedom equal to the difference in number of estimated parameters was used. Building on Model 2, we next added autism-related experience as second main effect:

$${\text{Model 2}}{\text{.1: EBP outcome}}\, \sim \,{1}\, + \,{\text{ICSTotal}}\, + \,{\text{ASD Exp}}\, + \,\left( {\text{1|District}} \right)\, + \,{\text{(1|SELPA)}}{.}$$

After investigating the main effects, we next examined how autism-related experience moderated the relationship between implementation climate and EBP use by including an interaction term between implementation climate and autism-related experience in the model. The model fitted is shown below:

$${\text{Model 2}}{\text{.2: EBP outcome}}\, \sim \,{1}\, + \,{\text{ICSTotal}}\, + \,{\text{ASD Exp}}\, + \,{\text{ICSTotal }} \times {\text{ ASD Exp}}\, + \,\left( {\text{1|District}} \right)\, + \,{\text{(1|SELPA)}}{.}$$

All other moderation analyses in this paper were conducted in this manner.

## Results

### Aim 1. Examine the Relationship Between Implementation Climate and Provider-Reported Outcomes of EBP Implementation

The total mean score of implementation climate across 333 districts was 38.89 (*SD* = 19.44). While scores are not normative, this is similar or slightly higher than other studies using the measure in educational settings (Lyon et al., 2018). The mean for EBP implementation was 2.56 out of 4 (*SD* = 0.68), indicating that on average, the EBP implementation outcome was rated somewhere between moderate to great. See Table 2 for all means and standard deviations. There was a significant relationship between the implementation climate and EBP implementation outcome (*χ*^*2*^ = 50.63, *df* = 1, *p* < 0.001). That is, the higher the implementation climate, the higher the EBP implementation outcome. Specifically, for every 1-point gain in implementation climate, there was a respective increase in EBP implementation outcome of 0.0092 (*SE* = 0.001, *t* = 7.28).

**Table 2:** Means and SDs of implementation climate, autism-related experience, EBP resources, EBP implementation outcome, and student outcomes

	Mean (SD)
Implementation climate	38.89 (19.44)
Focus on EBP	6.12 (2.87)
Educational support for EBP	5.05 (2.90)
Recognition of EBP	4.85 (2.64)
Rewards for EBP	2.39 (2.50)
Selection for EBP	4.76 (2.91)
Selection for openness	6.40 (2.60)
Existing supports	4.04 (2.90)
Use of data	5.28 (3.75)
Autism-related experience	2.36 (.64)
0 = Little to no hands-on/direct experience (%)	.6
1–2 = some experience (%)	36
3–4 = moderate to extensive (%)	63.4
EBP resources	41.41 (19.20)
EBP implementation outcomes	2.56 (.68)
Poverty rate	0.58 (.21)
Student outcomes at district level	
Math	.18 (.16)
English	.22 (.16)
LRE 80%	.40 (.20)
Separate placements	.04 (.04)
Suspension	.07 (.05)

### Aim 2. Examine Whether Provider Experience Moderates the Influence of Implementation Climate on EBP Implementation

On average, providers reported having moderate to extensive hands-on experience working with students with autism (*mean* = 2.36, *SD* = 0.64; see Table 2), and it was a significant predictor of EBP implementation outcome (*χ*^*2*^ = 105.78, *df* = 1, *p* < 0.001). That is, increased provider experience with autism was significantly related to EBP implementation outcome. For every 1-point increase in provider autism-related experience, there was an increase of 0.33 in EBP implementation outcome (*SE* = 0.03, *t* = 10.53).

**Fig. 1:**
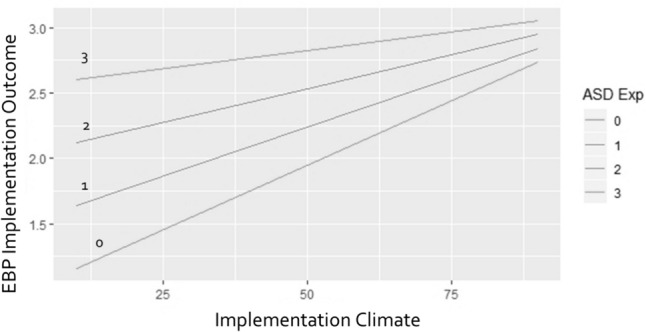
The moderation effect of provider autism-related experiences on implementation climate and EBP implementation outcomes

The result also supported a significant interaction effect between implementation climate and provider autism-related experience on EBP implementation outcomes (*χ*^*2*^ = 10.91, df = 1, *p* < 0.001). The level of implementation climate was more impactful on EBP implementation outcomes when provider’s hands-on autism experience was low. See Fig. 1.

### Exploratory Aim 3. Examine the Relationship Between EBP Resources and Student Outcomes in Districts with Varying Levels of Poverty

At the district level, total EBP resources scores ranged from 15 to 105 (*mean* = 41.41, *SD* = 19.20). District poverty level indicated by the percentage of socioeconomically disadvantaged students in the District ranged from 0.02 to 0.99 (*mean* = 0.58, *SD* = 0.21). In the 2018–2019 school year, several autistic student outcomes were examined. On average, 18% (*SD* = 0.16) of students with autism met state standards in math, 22% (*SD* = 0.16) met state standards in English, 40% (*SD* = 0.20) were in a regular education classroom for greater than 80% of the day, 4% (*SD* = 0.04) were placed in separate placements and 7% (*SD* = 0.05) of students with autism had suspensions within the given year.

**Fig. 2:**
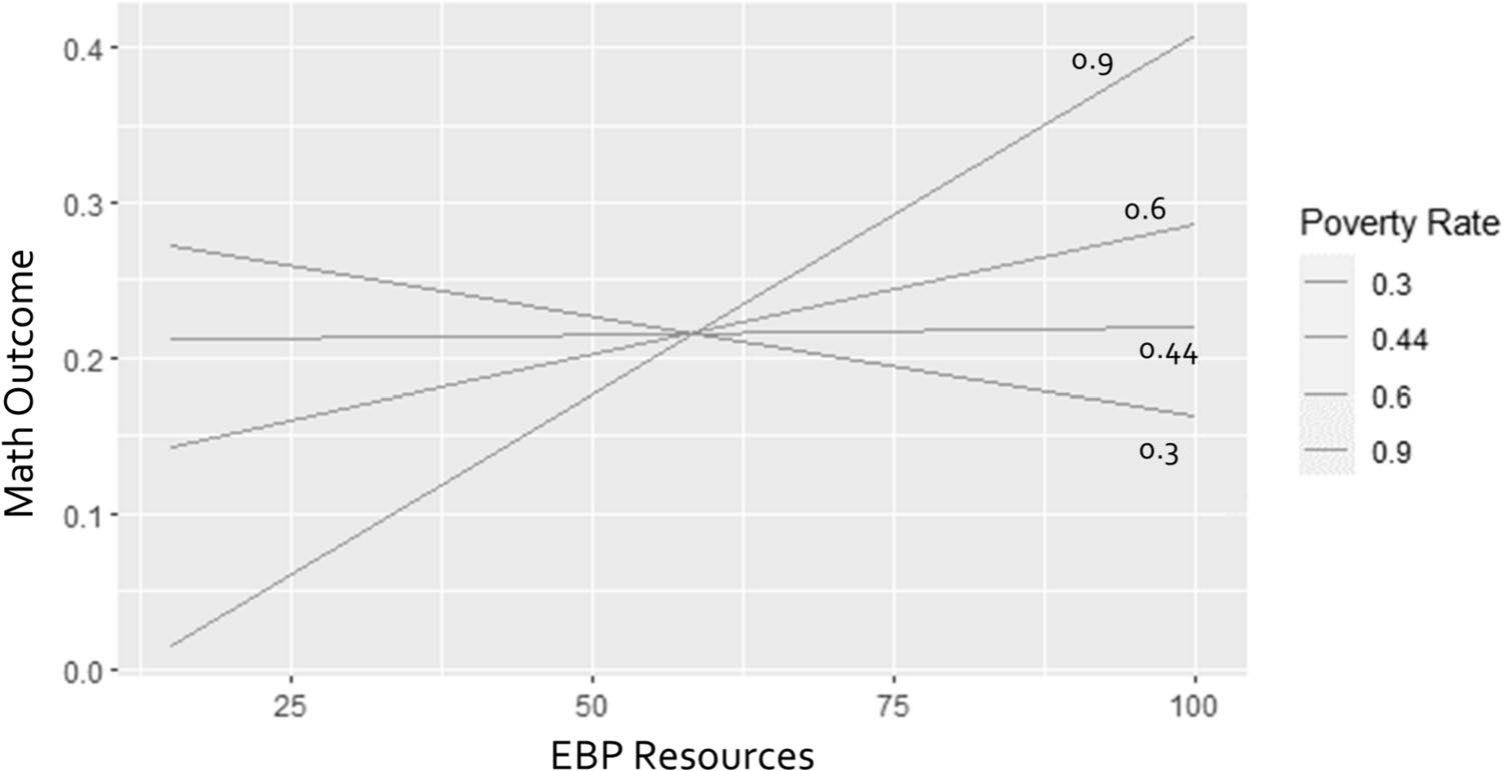
The moderation effect of district poverty rate on EBP resources and student math performance

The results showed that higher levels of EBP resources were related to better math achievement (*t* = 2.31, *SE* = 0.0009). A significant interaction between EBP resources and district poverty rate was found (*χ2* = 8.30, df = 1, *p* < 0.005). Higher levels of EBP resources predicted a higher percentage of students who met math standards only when the district poverty rate was high (*t* = 2.98, *SE* = 0.0036). That is, students from districts with high poverty rate may benefit further from increased EBP resources. See Fig. 2. No other interaction effects were found on other student outcomes.

## Discussion

The current study explored provider and system level moderating factors on provider EBP implementation and student outcomes. Data from a statewide survey of over 2000 education system administrators and educators serving autistic students in California provided a unique opportunity for examining moderation effects. Findings are relevant to the special education service system, but also contribute to the broader literature on how implementation factors may serve as moderators of child outcomes for autistic students.

Our two primary findings relate to the impact implementation climate and EBP resources have on the educator-reported implementation of the use of evidence-based autism practices. First, data indicate increased provider experience with autism was significantly related to EBP implementation outcomes, and the level of implementation climate was more impactful on EBP implementation outcomes when provider’s hands-on autism experience was low. This outcome directly informs possible organization-level implementation intervention. For example, when provider experience is low, targeted improvements to implementation climate may be helpful. Programs or school sites with providers new to the profession or new to autism services may benefit from employing strategies to enhance implementation climate. This is especially important for addressing disparities in service quality as schools located in areas with higher levels of poverty often have more newer teachers (Gagnon & Mattingly, 2012). Implementation climate might be improved through leadership training. A recent study examining the effects of leadership training to improve implementation leadership and climate found that, in schools and mental health clinics whose leaders received the leadership intervention, EBP fidelity and autistic student outcomes were greater compared to programs where leaders did not receive the training, and the intervention directly impacted implementation climate (Stahmer et al., 2022). This is consistent with other existing literature indicating the importance of implementation leadership on implementation climate and use of EBP for autism (Williams et al., 2022).

The second key finding from our exploratory analysis relates to the association between resource access and student outcomes. Although most distal outcomes were not associated with resources or EBP use, we did find some preliminary support for the need for increased resources in high poverty districts. Specifically, when school district poverty was high, higher levels of EBP resources were related to better math achievement for autistic students. This suggests targeted investment in resources to support autism EBP may be particularly beneficial for improving academic outcomes in high poverty school districts. Although EBP resources do require investment, there are ways for administrators to add EBP support through providing time for training and coaching, providing access to materials needed to implement the intervention, and having an intervention manual and training tools available which could be shared by multiple classrooms. In general, the findings from this work are consistent with other related literature which suggests added benefits of leadership behaviors directly targeting implementation of autism EBP. These outcomes are also consistent with other school-wide implementation findings about the importance of leadership when the intervention is not specific to autism. For example, in a district-wide scale up of Positive Behavior Intervention and Supports (PBIS), districts without supportive leadership and systems did not sustain (Kincaid & Horner, 2017; Horner et al., 2013). This alignment suggests implementation leadership as a key factor for school-based programming generally.

Together these findings suggest that providing implementation leadership training to district and school leaders may facilitate improved implementation of autism EBP and thereby improve learning for autistic students. Recent research supports leadership training as a successful implementation strategy to improve EBP implementation in community mental health clinics (e.g., Aarons et al., 2015; Williams et al., 2023). A recent implementation trial examining leadership training in public schools and publicly funded mental health clinics specifically serving autistic children found that in programs where leaders participated in brief leadership training providers had higher adherence to the EBP and those programs had better outcome for the participating autistic children (Stahmer et al., 2022). Multi-level leadership training could improve autism EBP use, increase teacher’s effective use and EBP and potentially improve student outcomes. This may be a cost-effective method of increasing access to high quality care for all autistic children.

In the current exploration, there are some limitations that should be noted. Our data were collected from a multidisciplinary group of educators and school-based providers in California. Participant perspectives and moderating factors may differ nationally within the US and internationally. Participant discipline and position may differentially affect perceptions of implementation climate and EBP use which has not been examined in these analyses. Additionally, data on implementation climate, EBP implementation and resources were all collected via self-report. This is reflective of how such constructs are measured in the field, but still involves the collective perspectives of participants. It should also be noted that overall poverty data are not specific to autistic students and may affect education for all students in the district. Finally, the sample, like the majority of educators in California, is primarily white and female and therefore may not be representative of more diverse areas.

In summary, this study explored system level moderating factors on provider EBP implementation and student outcomes, using a large sample of educators supporting students with autism. This is the first study of its kind, and identified specific mechanisms that may be targeted for system-level implementation intervention. Additionally, our findings suggest a direct path toward improving equitable access to EBP for autistic students.
