# Correction: The Role of Implementation Climate in Moderating Educator Use of Evidence-Based Practices and Outcomes for Autistic Students

**DOI:** 10.1007/s10803-024-06490-4

**Published:** 2024-07-23

**Authors:** Aubyn C. Stahmer, Yue Yu, Jessica Suhrheinrich, Melina Melgarejo, Patricia Schetter

**Affiliations:** 1 Department of Psychiatry and Behavioral Sciences, University of California Davis MIND Institute, Sacramento, CA USA; 2 Department of Special Education, San Diego State University, San Diego, CA USA; 3 Child and Adolescent Services Research Center, San Diego, CA USA; 4 California Autism Professional Training and Information Network (CAPTAIN), Sacramento, CA USA

**Correction: Journal of Autism and Developmental Disorders**

10.1007/s10803-024-06443-x

In this article the author name ‘Melgarejo’ was incorrectly written as ‘Melgajaro’.

The original article has been corrected.

